# Survival Outcomes of a Large Cohort of Acral Melanoma Patients Treated at a South African Referral Hospital

**DOI:** 10.1155/jskc/4779587

**Published:** 2025-01-23

**Authors:** Bianca Tod, Tonya Esterhuizen, Willem Visser, Maritha Kotze, Anne Bowcock, Johann Schneider, Henriette Burger

**Affiliations:** ^1^Division of Dermatology, Department of Medicine, Faculty of Medicine and Health Sciences, Stellenbosch University and Tygerberg Academic Hospital, Cape Town, South Africa; ^2^Division of Epidemiology and Biostatistics, Department of Global Health, Stellenbosch University, Cape Town, South Africa; ^3^Division of Chemical Pathology, Department of Pathology, Faculty of Medicine and Health Sciences, Tygerberg Academic Hospital, Stellenbosch University and National Health Laboratory Service, Cape Town, South Africa; ^4^Departments of Dermatology, Oncological Sciences and Genetics & Genome Science, Icahn School of Medicine at Mount Sinai, New York, New York, USA; ^5^Division of Anatomical Pathology, Department of Pathology, Faculty of Medicine and Health Sciences, Tygerberg Academic Hospital, Stellenbosch University and National Health Laboratory Service, Cape Town, South Africa; ^6^Division of Radiation Oncology, Faculty of Medicine and Health Sciences, Stellenbosch University and Tygerberg Academic Hospital, Cape Town, South Africa

## Abstract

Acral melanoma (AM) is the most common type of melanoma arising in people with skin of color. AM is often diagnosed late and associated with poor outcomes. Melanoma outcomes are also impacted by socioeconomic status. Although uncommon, AM is a public health concern in South Africa because of its epidemiology and association with health access issues, which predispose to late diagnosis. South African patients are managed based on staging systems and treatment guidelines developed for other populations. This cohort study aimed to determine the survival outcomes in a cohort of South African AM patients and how these were associated with demographic, clinical, pathological, and management data. The study included patients diagnosed at a referral hospital between 1 January 2010 and 31 December 2021. Analysis occurred in 2022 and 2023. The main outcomes of interest were survival time in months (overall and progression-free, OS and PFS). Survival outcomes were analyzed using Kaplan–Meier survival curves. Survival probabilities were compared between subgroups using log-rank tests. Univariate and multivariable analyses were performed using the Cox proportional hazards models to assess factors associated with survival. Ninety-one patients were included in the analysis. After a median follow-up of 28 months (range: 0–151 months), 48 patients (52.7%) had died. The 3- and 5-year survival rates for the entire cohort were 64.8% and 56.0% respectively. Notably, the OS and PFS were not affected by the population group (*p* value = 0.628, not significant). The examination of OS and PFS by the clinical stage group demonstrated proportional hazard. Although SLNB comprised a small group, the results appear to be prognostically valid, specifically for OS. The results indicate that the AJCC eighth edition staging is broadly applicable to AM in this population; however, a rigorous comparison was not possible. SLNB appears to be prognostically valid. No difference in survival outcomes by population group was shown.

## 1. Introduction

Acral melanoma (AM), defined here as melanoma arising on the palm, sole, or nail apparatus, is a unique subtype of malignant melanoma. It differs from other melanoma subtypes in terms of epidemiology, clinical and pathological features, prognosis, and molecular characteristics [1–3]. Our knowledge and understanding of AM have lagged in comparison to more common types of melanomas for a variety of reasons: delays in recognizing it as a unique entity, its relative rarity in developed societies where research is better supported, and differences in how it is defined (with some studies focusing on AM as defined here, others on the acral lentiginous melanoma (ALM) subset). Most reported AM cohorts have come from North America, Europe, and Asia, with only a few originating from Central and South America [1, 4–14]. A large proportion of the worldwide AM disease burden is therefore likely to be underrepresented, with a dearth of studies from Africa. This report presents the largest contemporary survival analysis of AM from an African institution [15–18]. It should be noted that there is no access to targeted or immunotherapy for melanoma in the public health system in South Africa (including the institution in this report). Stereotactic radiotherapy techniques are also not available at the institution yet. Stage IV disease is therefore always treated with palliative intent, even in the setting of oligometastatic disease. Also of note is the pervasive late presentation of patients with melanoma, which in practice means that many Stage III patients are present with advanced nodal metastases that are not resectable or that have rendered the patient unfit for aggressive surgery. The study aimed to determine overall survival (OS), progression-free survival (PFS), and survival probability (OS and PFS) and to analyze the relationship between clinical, pathological, and treatment variables and OS and PFS, in a group of South African AM patients. Of particular interest was the relationship between population group and survival outcomes, given the literature showing racial disparities in melanoma outcomes [19, 20].

## 2. Materials and Methods

Adult patients over the age of 18 years with a clinical and histological diagnosis of AM (melanoma arising on the palm, sole or nail apparatus), processed by the National Health Laboratory Service (NHLS) at Tygerberg Academic Hospital (TAH) between 1 January 2010 and 31 December 2021 were included in this retrospective study. The clinical and pathological features of a large proportion of these patients have been reported previously [21].

Exclusions were as follows:

• Melanomas not involving the palm, sole, or nail apparatus (subungual), i.e., not meeting the criteria for AM.
• Patients with recurrent AM, initially diagnosed before the study period.
• Patients without an available South African identity number and only a single clinical contact time point in the available databases.

Clinicopathological data were extracted by B.T. and H.B. from individual patient records and from the hospital's electronic clinical and radiological records' systems, as well as the electronic NHLS records' system (both histological and other laboratory reports). Survival data were obtained by querying the Department of Home Affairs Death Register through the South African Medical Research Council (SAMRC) National Population Register. Dates of death were confirmed with hospital records where possible. Data on the cause of death were not consistently available and therefore all-cause mortality is reported here.

Age was defined as completed years between the date of birth and the date of histological diagnosis. Population group (using categories from the South African census) was used as a proxy for skin color^∗^, as Fitzpatrick phototype was only collected routinely towards the end of the study period. Where the population group was not recorded in the clinical notes, it was determined through a validated imputation method [22]. Diagnostic delay was defined as completed months from the month when the patient first reported noting the lesion to the date of histological diagnosis. This period encompasses both patient and healthcare system delays. It is acknowledged that there is an inherent subjectivity and unreliability to this measure.

Histological features such as Breslow thickness (BT) and mitotic rate were captured as the highest measurement recorded amongst the initial histological specimens (for example, if the initial incisional biopsy demonstrated a mitotic rate of 3/mm^2^ and the wide local excision (WLE) demonstrated a mitotic rate of 1/mm^2^, the mitotic rate was recorded as 3/mm^2^). BT and mitotic rate were divided into clinically relevant categories for statistical analysis.

The clinical stage was determined using the American Joint Cancer Committee (AJCC) eighth edition Tumor, Node, Metastasis (TNM) classification [23]. A composite stage group designation was used (presented here as “clinical stage”) to incorporate as much data from the initial battery of investigations and render as accurate a biological stage at presentation as possible, and to render the highest proportion of patients stageable. If patients had no clinical evidence of regional or distant disease on examination and no further confirmatory imaging was undertaken, they were considered to have localized disease [24]. Patients with unknown nodal status were designated Nx as described by AJCC. In some cases, patients could be designated as having nodal disease, but this could not be more accurately staged (e.g., the patient had a positive fine needle aspirate but no further staging investigations). These patients were designated as “positive but unable to stage further.”

The study period is defined as the time from the first patient's histological diagnosis (time point 0, baseline) to the date of data censoring. OS was defined as the time in months between the date of histological diagnosis and the date of death from any cause for deceased patients. Censoring of patients known to be alive occurred on 31 May 2022 for patients with South African identity document numbers (IDNs), or the date last seen for clinical follow-up for patients without IDN. PFS was defined as the time in months between the date of histological diagnosis and the date of clinical, radiological, or histological progression, relapse, or death, whichever came first. Progression was defined as a new disease detected less than a year after treatment, while relapse was defined as a new disease detected more than a year after treatment. Management intent was captured as either curative (radical) or palliative.

Data were captured in an Excel spreadsheet and analyzed using SPSS Version 28. Descriptive statistics were used to present clinicopathological data. Survival outcomes (including OS, PFS, and 3- and 5-year survival rates) were analyzed using Kaplan–Meier survival curves. Survival probabilities were compared between subgroups using log-rank tests. Chi-squared 2-sided exact tests were used to assess associations between clinicopathological risk factors and 3- and 5-year survival rates. Univariate and multivariable analyses to assess factors associated with OS and PFS were performed using the Cox proportional hazards models. Variables where 40% or more cases had missing values were excluded from multivariable analyses. Independent variables with univariate *p* values of < 0.2 were considered in the multivariable model. A value of < 0.2 was selected to indicate the possible importance of the predictor variable rather than statistical significance. This practice has the advantage of screening in possible associations due to confounding which might otherwise be missed if a stricter cut-off is used at this stage. However, for the multivariable analysis, a *p* value < 0.05 indicated statistical significance. A backwards stepwise method was used to arrive at the final model with entry and exit probabilities set at 0.05 and 0.1, respectively. A *p* value of < 0.05 was considered statistically significant.

Ethical clearance for this project was obtained from the Stellenbosch University Health Research Ethics Committee (N15/12/129).

## 3. Results

### 3.1. Demographic, Clinical, and Pathological Data

A total of 91 patients were included in the analysis. The median age at diagnosis was 59 years (range 26–88 years). Data on the Fitzpatrick phototype were available for 33 patients (36.3%) and thus only summarized descriptively. The median treatment delay was 2.9 months (range 0–96.3 months) and ascertainable in 68 (74.7%) patients. Other categorical descriptive statistics are summarized in Table 1.

### 3.2. Survival Probabilities

#### 3.2.1. OS of 91 Patients

**Figure 1:**
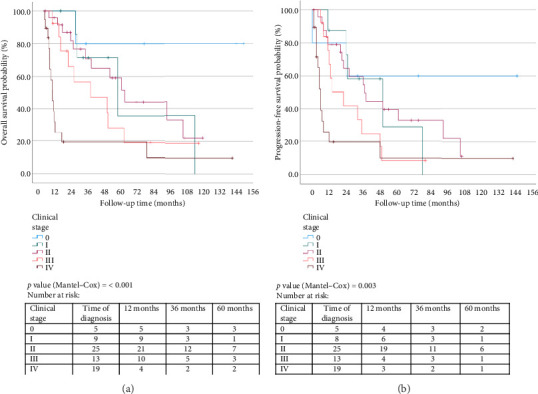
Kaplan–Meier survival curves by AJCC eighth edition clinical stage (a) overall survival and (b) progression-free survival.

After a median follow-up of 28 months (range: 0–151 months), 48 patients (52.7%) had died. The median OS for the entire cohort was 55.9 months (95% CI: 38.5–73.3 months) and 48.1 months (95% CI: 24.5–71.7 months) when only the patients with the composite stage group determination were analyzed (Table 2). The 3- and 5-year survival rates for the entire cohort were 64.8% and 56.0%, respectively, and 60.6% and 52.1% for the group with the composite stage group assigned. There was a statistically significant difference in OS probability and 3-year survival rate by composite stage (*p* values < 0.001 and 0.02) as shown in Table 2 and Figure 1. The Stage IV patient with a remarkably long survival time was staged based on abdominal sonar and CT scan which demonstrated liver masses consistent with metastases at the time of primary diagnosis. Soon after, the patient was lost to follow-up. This patient probably did not have Stage IV disease at diagnosis, considering her long survival time. The other Stage IV patient with long survival experienced such long delays in her primary staging that more than 2 years elapsed between her first biopsy and her staging PET scan. Presumably, she did not have Stage IV disease at the time of diagnosis, although this could not be proven.

#### 3.2.2. PFS of 90 Patients

PFS was determined for 90 patients as the date of progression of one patient was missing. By the end of the follow-up period, 56 (62.2%) patients had experienced progression, recurrence, and/or died. During the study period, seven patients (7.7%) had a confirmed recurrence, and 18 experienced disease progression (19.8%). Median PFS time was 35.0 months (21.5–48.5).

### 3.3. Factors Associated With Survival

#### 3.3.1. OS of 91 Patients

Univariate analysis revealed an association between reduced OS and older age, nail location, nodular or NOS histological subtypes, presence of ulceration, mitotic rate greater than 3/mm^2^ (both the > 3–10/mm^2^ and > 10/mm^2^ groups), nodal status N2, N3, or positive but unable to stage further, presentation in stage IV, and the absence of surgical intervention (*p* < 0.05, see Tables 3a and 3b). BT (when assessed as a continuous variable) was associated with OS, with each additional mm of BT resulting in a 7% higher hazard of mortality. Multivariable modeling showed that age, an unknown histological subtype, BT, a positive sentinel lymph node biopsy, or if it was not performed, and nodal positivity (specifically N2 and N3 statuses) were independent poor prognostic factors for OS. Protective factors included unknown nodal status and undergoing surgical treatment (*p* < 0.05, hazard ratios presented in Tables 3a and 3b). The counter-intuitive protective effect of increasing mitotic rate in the multivariable model was explored statistically (basic cross-tabulation with OS) and by examining the effect of step-by-step introduction of variables into the model. The increasing mitotic rate category was associated with reduced OS in the univariate analysis, and distortion of the hazard ratios in the multivariable model did not occur due to interaction with any single variable. This finding is likely due to confounding and should be interpreted with caution.

#### 3.3.2. PFS of 90 Patients

Univariate analysis demonstrated an association between PFS and histological subtype (specifically unknown or NOS subtypes), nodal status positive without details, N2 and N3, distant metastases on PET scan, and Stage IV disease having poor prognostic implications (*p* < 0.05), (see Tables 3a and 3b). Multivariable modeling revealed that histological subtype (specifically the NOS subtype), nodal status “positive without details,” and N2 were significant predictors of progression (including death) (*p* < 0.05). Only surgical treatment was protective for progression (see Tables 3a and 3b).

#### 3.3.3. Analysis of Subgroups of Interest

Analysis of outcomes (OS and PFS) by BT, ulceration, and mitotic rate, respectively, in patients from Stages I and II did not reveal a trend. Amongst patients who had SLNB performed (*n* = 15), OS was 16.7% for those with positive SLNB, and 66.7% for those with negative SLNB (*p*-value = 0.119). Management intent (radical vs. palliative) amongst Stage III patients did not influence OS or PFS.

## 4. Discussion

This AM cohort demonstrated a 3-year OS of 64.8% and a 5-year OS of 56.0%, and a median survival time of 55.9 months (38.5–73.3). Most of the findings in this study were consistent with findings in AM in other populations. The authors, however, acknowledge that any comparison of the current survival outcomes with that of other published cohorts should be performed with caution due to variations in definitions, treatment approaches, outcomes reported, and missing data (for example, AM vs. ALM, OS vs. melanoma-specific survival [MSS]). The absence of an association between the population group and survival outcomes in the current study is, however, notable, and an important difference from data reported in other populations [19, 20]. The prognostic value of SLNB as demonstrated in this study may have significant implications for the management of patients with AM in a resource-constrained environment.

Our results are in line with the 57% 5-year OS reported in a Texan institutional cohort of 583 patients with ALM [25]. Their data presumably predate the widespread use of modern systemic therapies (1999–2014), which reflects the current state of melanoma management at our institution. The modest survival benefit associated with the ALM subtype in the current cohort necessitates reserved comparison between the studies (see Tables 3a and 3b). An English registry-based 5-year OS for subungual melanoma (1984–1993) was 51% [26]. MSS rates are higher than OS as expected. A multicenter study of ALM in the US reported a 5-year MSS of 78.1% [5].

From a quality-of-life perspective, the median PFS (here defined as including relapse and progression) was 35.0 months. A median recurrence-free survival of 24.1 months was reported in a Turkish study of 102 ALMs [27]. The examination of OS and PFS by the clinical stage group demonstrated proportional hazard, which is consistent with the AJCC eighth edition staging being broadly applicable to AM in this context. This is important as the use of AJCC eighth edition has not been validated for the staging of AM patients in South Africa, even though it is used. It must be cautioned that a rigorous comparison with AJCC outcomes (prognoses) was not possible, as many patients did not receive appropriate treatment, and there were large amounts of missing data. Univariate analysis did not reveal an unexpectedly prominent role for BT, ulceration, or mitotic rate, within the Stage I and II groups. The clinical and pathological features of this cohort of AMs were consistent with those reported previously [1, 21, 28]. In terms of OS, there was a slight increase in mortality with each year of age and in males.

The population group distribution correlated with the general patient demographics in the drainage area of the TAH, in stark contrast to melanoma of all types that affects mainly White patients [29]. Notably, the OS and PFS were not affected by population group (*p* value = 0.628, not significant^1^). Many previous studies from other countries have demonstrated disproportionately better survival of White patients with melanoma [19, 30]. The current study did not find this effect, probably due to the relatively small cohort, or the cohort being drawn from a single institution with relatively consistent diagnosis and management of patients from a low-income population. This could imply that equal access to healthcare and standardized treatment protocols would correct for disparities in survival outcomes reported elsewhere. A multi-institutional South African study examining melanoma outcomes would give a more accurate result.

Whilst not carried through to the multivariable model, the univariate analysis of anatomical location correlated strongly with OS. A lesser but similar effect was seen for PFS. Subungual location was associated with particularly poor outcomes (HR = 12.1, 95% CI: 1.5–99.8, *p* value = 0.020), consistent with an Australian study but in direct contrast to a Korean study [31, 32]. These conflicting results suggest that other factors contribute to poor prognosis in different geographical contexts, rather than subungual location being an independent prognostic factor [25].

OS and PFS also correlated with the histological subtype of AM on univariate analysis. Nodular subtypes were associated with worse outcomes, but lesions coded NOS had significantly worse outcomes on univariate analysis. This finding only carried through to the multivariable model for PFS (and was statistically significant, *p* value = 0.022). Lesions with unknown histological subtypes also had a poor outcome. This finding may be attributed to confounding factors as advanced melanomas were more likely classified as NOS or unknown subtypes.

The perceived difference in the outcomes between ALM and non-ALM AM remains to be confirmed. Considering the association of *KIT* alterations with the lentiginous growth pattern, important biological differences might be at play [33]. These and other features could have important treatment implications, with regards to the use of targeted therapy. For example, a study by Moon et al. demonstrated that *BRAF* mutations are associated with epithelioid cell types [34]. These kinds of observations could ultimately direct more selective tumor genetic testing, based on pathology and druggable targets.

Univariate analysis did not reveal a prominent prognostic role for BT, ulceration, or mitotic rate within the Stage I and II groups. This is most likely due to the small dataset and the advanced stage of the lesions (for example, 52.7% had BT > 4 mm). BT correlated with OS, with each additional mm of BT resulting in a 7% higher likelihood of mortality. Although this finding appeared to be the trend in the categorical (T1-4) classification of BT, it did not reach statistical significance. BT is considered the main determinant of prognosis in localized melanoma; however, the discrepancy between the small number of tumors with BT ≤ 2 mm and the large number of patients with BT > 2 mm may explain the lack of statistical significance [35]. Ulceration did indicate poor prognosis on the univariate analysis of OS; however, this was not reflected in the multivariate model or PFS. Ulceration was confirmed as an adverse prognostic feature in a much larger study of ALM [36]. A mitotic rate greater than 3/mm^2^ had an adverse prognostic value for OS and PFS, although statistical significance was not always reached. There was a particularly high incidence of AMs with mitotic rates of greater than 3/mm^2^ (35.2%). This observation is difficult to compare to other cohorts due to differences in reporting parameters.

The study found limited use but significant prognostic value in SLNB. SLNB was only performed in 15 patients (16.5%), of whom 6 were positive. Although SLNB comprised a small group, the results appear to be prognostically valid, specifically for OS. Overall, nodal Stages N2 and N3 were shown to be a strong indicator of prognosis for OS and PFS on univariate and multivariable models. Both the N1 and Nx groups had nonsignificant *p* values. The N1 group included 5 N1a patients (out of 11), which may have skewed the prognostic effect of the N1 stage as it combined a low-risk group (N1a) with patients who have clinically detectable nodes or in-transit, satellite, and/or microsatellite metastases. The Nx group presumably included patients of all nodal stages and was heterogenous. It is important to better understand the value of SLNB in resource-constrained environments, where patients do not have access to novel therapies such as immunotherapy or targeted therapies, and where the economic and personal implications of each additional procedure differ from those in better-resourced settings.

Surgical treatment offered an OS and PFS advantage to patients, both in the univariate and multivariable models, as found in a recent Korean study [37]. This finding is important in the South African setting where the costs and benefits of surgery need to be carefully considered. It is also important in a condition where surgical management may involve amputation, an outcome associated with significant morbidity. Within the Stage III group in this cohort, management intent (radical vs. palliative) did not influence outcomes. Patients with Stage III melanoma could benefit from targeted or immunotherapies. The implications for AM patients are less clear as responses to advanced therapies such as immunotherapy in AM patients are not as impressive [38]. A better understanding of the genetic drivers of AM in this context may shed light on the potential value of targeted therapies in AM patients. These findings could then be entered into the pipeline of patient diagnosis, subtyping, and ultimately treatment. This approach relates to pathology-supported genetic testing (PGST), recently validated as a pharmacodiagnostic tool [39]. PGST links individual characteristics and precision treatments.

The generalizability of this study was limited by its retrospective nature, the proportion of missing data, and by small patient numbers (especially for subgroup analysis). Furthermore, many studies focus on the ALM subtype; however, this study includes all histological subtypes. This may have compromised the homogeneity of the cohort. An analysis of MSS would have been ideal; however, these data are not reliably available in our context. The analysis of PFS mostly reflected patients with progression, rather than relapse. The OS data are more reliable as an objective database was used, as opposed to clinical notes. The start date used to define PFS in this study is unconventional, as this parameter is typically used in interventional studies and is defined by the date of initiation of therapy. PFS was included as part of this small observational study (where a large proportion of patients did not undergo treatment) to give a more complete representation of the cohort's outcomes. The comparison of outcomes to those predicted by AJCC's eighth edition must be viewed with some caution as a large proportion of patients did not receive “standard of care” treatment, or there was missing data relating to their treatment. Despite these limitations, this study adds important data to a poorly understood and underresearched disease in Africa.

Recommendations based on the findings in this study include the recognition of AM as a disease priority in the South African context (it is uncommon but there are significant potential gains from improving management). There is a need for improved awareness to ensure early diagnosis, since most patients presented with T4 tumors. System failures in the care rendered to AM patients need to be addressed with urgency.

## 5. Conclusions

Analysis of this cohort of AM patients did not reveal any findings that were inconsistent with previous cohorts from other countries. These results demonstrate that the AJCC staging system probably applies in this context; however, there are limitations to this conclusion. SLNB offers reliable prognostic information (albeit based on a small group) in this clinical context, and surgical management offers both OS and PFS benefits. Although not statistically significant, the population group did not adversely affect the outcomes for this small cohort. Further investigation of South African AM patients is critical to confirm these findings, as the guidelines used to treat them were not informed by research on our population. More importantly, to improve patient outcomes, strategies to increase AM awareness should be prioritized. The value of this study is the relative underrepresentation of such patients in the literature and the opportunities for the improvement of such patients' outcomes, which emphasizes the need for further research specific to African populations.

## Figures and Tables

**Table 1:** Summary of categorical characteristics of 91 AM patients included in this study.

Characteristic		*N*	%
Sex	Female	58	63.7
	Male	33	36.3

Population group	Black African	45	49.5
	Mixed ancestry	32	35.2
	White	13	14.3
	Asian/Indian	1	1.1

Fitzpatrick phototype	I	3	3.3
	II	0	0
	III	3	3.3
	IV	13	14.3
	V	9	9.9
	VI	5	5.5
	Unknown	58	63.7

Anatomical location	Sole	75	82.4
	Palm	7	7.7
	Nail apparatus	9	9.9

Histological subtype	ALM	39	42.9
	Superficial spreading	3	3.3
	Nodular	11	12.1
	NOS	19	20.9
	Unknown	19	20.9

Tumor (T) category	Tis	5	5.5
	T1	4	4.4
	T2	10	11.0
	T3	14	15.4
	T4	48	52.7
	Measurement not possible^∗^	10	11.0

Ulceration	Yes	65	71.4
	No	20	22.0
	Unknown	6	6.6

Mitotic rate (/mm^2^)	None identified	8	8.8
	1–3	37	40.7
	4–10	25	27.5
	> 10	7	7.7
	Unknown	14	15.4

Histologic regression^∗∗^	Not identified	45	49.5
	Present	14	15.4
	Not reported	32	35.2

Sentinel lymph node biopsy (SLNB) result	Negative for melanoma	9	9.9
	Positive for melanoma (5 N1a and 1N2a)	6	6.6
	Not indicated	76	83.5

Clinical nodal status (N)	N0	35	38.5
	N1	11	12.1
	N2	4	4.4
	N3	10	11.0
	NX	27	29.7
	Positive but unable to stage further	4	4.4

PET scan result	Negative	6	6.6
	Regional nodes	8	8.8
	Distant metastases	10	11.0
	Abnormal but unknown significance	8	8.8
	Not indicated	59	64.8

Composite clinical stage at presentation	0	5	5.5
	1	9	9.9
	2	25	27.5
	3	13	14.3
	4	19	20.9
	Incomplete	20	22.0

Treatment intent	Radical (curative)	44	48.6
	Palliative	26	28.6
	Unknown	21	23.1

Treatment type	Surgery only	48	52.7
	Radiotherapy only	14	15.4
	Surgery and radiotherapy	3	3.3
	Other	7	7.7
	Unknown	19	20.9

Surgical procedure	Wide local excision (WLE)	30	33.0
	WLE + SLNB	14	15.4
	WLE + complete lymph node dissection	5	5.5
	Debulking with positive margins	2	2.2
	Not applicable	40	44.0

^∗^Typically, histological specimens consisting of only tumor and no normal tissue that could not be orientated for BT measurement. This usually occurs in the case of very large tumors.

^∗∗^Histologic regression, defined as the presence of a lymphohistiocytic infiltrate which replaces tumor cells, epidermal attenuation, dermal fibrosis and inflammatory cells, melanophagocytosis, and telangiectasia [23].

**Table 2:** Survival outcomes by stage (excludes patients with incomplete staging).

Clinical stage	Median OS (months) (95% CI)	3-year overall survival (%)	*p* value	5-year overall survival (%)	*p* value	Median PFS (months) (95% CI)
0	Not reached	80.0	**0.020**	80.0	0.096	Not reached
1	55.9 (11.3–100.4)	77.8		66.7		50.0 (12.0–87.9)
2	61.2 (42.8–79.6)	76.0		64.0		37.0 (18.2–54.2)
3	35.5 (0–74.7)	53.8		38.5		22.0 (7.0–36.9)
4	6.8 (4.2–9.4)	31.6		31.6		6.0 (4.2–7.8)
Overall	48.1 (24.5–71.7)	60.6		52.1		24.0 (12.9–35.0)

*Note:* Values in bold are statistically significant.

**(a):** 

		Univariate analysis (*n* = 91)				Multivariable analysis (*n* = 86)	
		*n*	Cox proportional hazards		Kaplan–Meier log-rank test *p* value	Cox proportional hazards	
			Hazard ratio (95% CI)	*p* value		Hazard ratio (95% CI)	*p* value
Age at diagnosis		91	1.024 (1.000–1.048)	**0.049**	—	1.089 (1.047–1.134)	**< 0.001**

Sex	Female	58	1	—	0.141	—	—
	Male	33	1.568 (0.857–2.869)	0.144		—	—

Population group	Black African	45	1	—	0.628	—	—
	White	13	1.277 (0.583–2.797)	0.541		—	—
	Mixed ancestry	32	1.011 (0.538–1.900)	0.974		—	—
	Asian/Indian	1	(Not computed)	—		—	—

Fitzpatrick phototype	I and II (II *n* = 0)	3	1	—	0.414	—	—
	III and IV	16	1.503 (0.185–12.234)	0.703		—	—
	V and VI	14	2.575 (0.316–20.974)	0.377		—	—
	Unknown	58	1.279 (0.173–9.470)	0.809		—	—

Anatomical location	Palm	7	1	—	**0.025**	—	—
	Sole	75	6.940 (0.949–50.731)	0.056		—	—
	Nail apparatus	9	12.125 (1.474–99.759)	**0.020**		—	—

Histological subtype	ALM	39	1	—	**< 0.001**	1	—
	Superficial spreading	3	(Not computed)	—		(Not computed)	—
	Nodular	11	2.572 (1.124–5.887)	**0.025**		3.475 (0.788–15.319)	0.100
	NOS	19	4.874 (2.108–11.269)	**< 0.001**		1.864 (0.513–6.769)	0.344
	Unknown	19	1.851 (0.874–3.918)	0.108		8.392 (2.081–33.843)	**0.003**

Breslow thickness	≤ 1 mm	4	1	—	0.126	1	—
	> 1–2 mm	10	0.533 (0.089–3.197)	0.491		0.248 (0.009–6.762)	0.409
	> 2–4 mm	14	1.325 (0.274–6.412)	0.727		0.126 (0.018–0.875)	**0.036**
	> 4 mm	48	1.847 (0.439–7.775)	0.403		7.131 (1.255–40.523)	**0.027**
	Not possible	10	1.784 (0.359–8.856)	0.479		2.494 (0.713–8.718)	0.152

Ulceration	No	20	1	—	0.071	—	—
	Yes	65	2.353 (1.079–5.129)	**0.031**		—	—
	Unknown	6	1.211 (0.256–5.731)	0.810		—	—

Mitotic rate (/mm^2^)	None identified	8	1	—	**0.007**	1	—
	One-three	37	1.273 (0.426–3.801)	0.666		0.070 (0.005–0.930)	**0.044**
	> 3–10	25	3.287 (1.076–10.042)	**0.037**		0.352 (0.028–4.477)	0.421
	> 10	7	3.988 (1.045–15.213)	**0.043**		0.692 (0.042–11.429)	0.797
	Unknown	14	1.022 (0.274–3.820)	0.974		0.042 (0.002–0.742)	**0.031**

Histologic regression	Not identified	45	1	—	0.522	—	—
	Present	14	0.745 (0.320–1.734)	0.494		—	—
	Unknown	32	1.231 (0.660–2.297)	0.513		—	—

SLNB result	Negative	9	1	—	0.259	1	—
	Positive	6	3.231 (0.771–13.545)	0.109		149.777 (7.398–3032.343)	**0.001**
	Not performed	76	2.048 (0.631–6.646)	0.232		17.134 (2.305–127.359)	**0.006**

Nodal status (N)	N0	35	1	—	**< 0.001**	1	—
	N1	11	2.005 (0.804–4.999)	0.136		0.729 (0.181–2.938)	0.656
	N2	4	15.189 (4.567–50.521)	**< 0.001**		29.782 (4.850–182.870)	**< 0.001**
	N3	10	7.438 (2.949–18.757)	**< 0.001**		23.797 (4.394–128.876)	**< 0.001**
	NX	27	0.966 (0.444–2.099)	0.930		0.092 (0.023–0.374)	**< 0.001**
	Positive but unable to stage further	4	4.543 (1.270–16.252)	**0.020**		1.431 (0.207–9.888)	0.717

PET scan result (if done)	Negative	6	1	—	**0.007**	1	—
	Regional nodes	8	0.753 (0.187–3.028)	0.689		0.246 (0.038–1.579)	0.139
	Distant metastases	10	3.248 (0.933–11.312)	0.064		0.659 (0.074–5.881)	0.708
	Abnormal but unknown significance	8	0.571 (0.127–2.567)	0.465		0.163 (0.020–1.302)	0.087

Clinical stage at presentation	0	5	(Not computed)	—	**< 0.001**	—	—
	1	9	1	—		—	—
	2	25	1.011 (0.325–3.144)	0.985		—	—
	3	13	1.680 (0.516–5.464)	0.389		—	—
	4	19	4.242 (1.377–13.062)	**0.012**		—	—
	Incomplete	20	0.667 (0.199–2.230)	0.667		—	—

Treatment delay		67	0.982 (0.946–1.019)	0.328	—	—	—

Surgical treatment	No	35	1	—	**0.049**	1	—
	Yes	51	0.482 (0.265–0.877)	**0.017**		0.251 (0.106–0.593)	**0.002**
	Unknown	5	0.806 (0.239–2.726)	0.729		0.789 (0.149–4.167)	0.780

**(b):** 

		Univariate analysis (*n* = 90)				Multivariable analysis (*n* = 85)	
		Cox proportional hazards			Kaplan–Meier log-rank test *p* value	Cox proportional hazards	
		*n*	Hazard ratio (95% CI)	*p* value		Hazard ratio (95% CI)	*p* value
Age at diagnosis		90	1.013 (0.991–1.036)	0.242	—	—	—

Sex	Female	57	1	—	0.714	—	—
	Male	33	1.110 (0.636–1.935)	0.714		—	—

Population group	Black African	45	1	—	0.450	—	—
	White	13	1.471 (0.702–3.083)	0.306		—	—
	Mixed ancestry	31	1.128 (0.632–2.014)	0.685		—	—
	Asian/Indian	1	(Not computed)	—		—	—

Fitzpatrick phototype	I and II (II *n* = 0)	3	1		0.380	—	—
	III and IV	15	1.859 (0.235–14.684)	0.557		—	—
	V and VI	14	2.800 (0.354–22.130)	0.329		—	—
	Unknown	58	1.519 (0.207–11.129)	0.681		—	—

Anatomical location	Palm	7	1	—	0.091	—	—
	Sole	74	3.963 (0.958–16.401)	0.057		—	—
	Nail apparatus	9	4.921 (1.014–23.876)	0.048		—	—

Histological subtype	ALM	39	1		**< 0.001**	1	—
	Superficial spreading	3	(Not computed)	—		—	—
	Nodular	11	2.169 (0.964–4.880)	0.061		1.765 (0.629–4.953)	0.280
	NOS	18	6.198 (2.865–13.411)	**< 0.001**		3.597 (1.207–10.718)	**0.022**
	Unknown	19	2.260 (1.134–4.505)	**0.020**		1.813 (0.807–4.075)	0.150

Breslow thickness (categorical)	≤ 1 mm	4	1	—	0.081		
						—	
	> 1–2 mm	9	0.558 (0.093–3.347)	0.523		—	
	> 2–4 mm	14	1.568 (0.332–7.406)	0.570		—	
	> 4 mm	48	2.314 (0.552–9.700)	0.251		—	
	Not possible	10	2.482 (0.526–11.721)	0.251		—	

Ulceration	No	20	1	—	0.151	—	
	Yes	64	1.973 (0.981–3.969)	0.057		—	
	Unknown	6	1.839 (0.505–6.694)	0.355		—	

Mitotic rate (/mm^2^)	None identified	8	1	0.074	0.056	1	—
	1–3	36	1.036 (0.390–2.757)	0.943		0.649 (0.145–2.904)	0.572
	> 3–10	25	1.944 (0.708–5.339)	0.197		0.980 (0.202–4.750)	0.980
	> 10	7	2.967 (0.890–9.893)	0.077		1.758 (0.286–10.812)	0.542
	Unknown	14	0.894 (0.283–2.821)	0.849		0.305 (0.052–1.799)	0.190

Histologic regression	Not identified	45	1	—	0.101	—	
	Present	14	0.544 (0.237–1.250)	0.152		—	
	Unknown	31	1.349 (0.763–2.387)	0.303		—	

SLNB result	Negative	9	1	—	0.162	—	
	Positive	6	3.276 (0.781–13.740)	0.105		—	
	Not performed	75	2.877 (0.894–9.257)	0.076		—	

Nodal status (N)	N0	34	1	—	**< 0.001**	1	—
	N1	11	1.255 (0.530–2.973)	0.606		1.368 (0.493–3.796)	0.547
	N2	4	9.376 (2.969–29.607)	**< 0.001**		7.630 (1.878–30.995)	**0.004**
	N3	10	6.019 (2.406–15.056)	**< 0.001**		2.557 (0.824–7.931)	0.104
	NX	27	0.721 (0.355–1.465)	0.366		0.576 (0.229–1.446)	0.240
	Positive but unable to stage further	4	3.110 (1.049–9.224)	**0.041**		3.938 (1.033–15.014)	**0.045**

PET scan result (if done)	Negative	6	1	—	**0.014**	—	
	Regional nodes	8	1.559 (0.438–5.550)	0.494		—	
	Distant metastases	10	3.775 (1.082–13.170)	**0.037**		—	
	Abnormal but unknown significance	8	1.096 (0.294–4.093)	0.891		—	

Clinical stage at presentation	0	5	(Not computed)	—	**0.003**		
	1	8	1	—		—	
	2	25	1.004 (0.366–2.750)	0.995		—	
	3	13	1.796 (0.623–5.179)	0.279		—	
	4	19	3.395 (1.203–9.578)	**0.021**		—	
	Incomplete	20	0.476 (0.154–1.471)				

Treatment delay		66	0.980 (0.946–1.016)	0.267	—	—	

Surgical treatment	No	34	1	—	**0.001**	1	—
	Yes	51	0.368 (0.210–0.646)	**< 0.001**		0.239 (0.114–0.503)	**< 0.001**
	Unknown	5	0.521 (0.155–1.747)	0.291		0.409 (0.086–1.948)	0.239

*Note:* Values in bold are statistically significant.

## Data Availability

De-identified data are available from the first author on request, with approval from the local ethics committee.

## Nomenclature

AJCC: American Joint Cancer Committee
ALM: Acral lentiginous melanoma
AM: Acral melanoma
BT: Breslow thickness
CI: Confidence interval
HR: Hazard ratio
IDN: Identity document number
M: Metastatic category
MSS: Melanoma-specific survival
N: Nodal category
NOS: Not otherwise specified
OS: Overall survival
PFS: Progression-free survival
SAMRC: South African Medical Research Council
SLNB: Sentinel lymph node biopsy
T: Tumor category
TNM: Tumor, Node, Metastasis classification
WLE: Wide local excision

## Endnotes

^1^The reporting of the population group was included as many previous studies on melanoma have reported differences in survival outcomes between individuals categorized in different population groups. Individuals with different skin colors may also be predisposed to melanoma via different genetic pathways, due to the protective effect of melanin.
